# A qualitative analysis of STEM female’s coping strategy under the COVID-19 pandemic

**DOI:** 10.3389/fpubh.2023.1298619

**Published:** 2023-11-23

**Authors:** Hsiu-Lan Shelley Tien, Lei Gong, Wei-Hsuan Wang, James Lee

**Affiliations:** Department of Educational Psychology and Counseling, National Taiwan Normal University, Taipei City, Taiwan

**Keywords:** COVID-19, career coping strategy, STEM, female, qualitative research

## Abstract

The purpose of the research was to realize the STEM female’s career coping under the pandemic. We conducted in-depth interviews with three STEM female engineers in Silicon Valley, California. After analyzing the research results, we found that: (1) In response to the impact of the pandemic, technology companies and female workers have demonstrated their ability to respond quickly; (2) While working from home, STEM females experienced five notable challenges, but also developed corresponding coping strategies; (3) Corporate systems and teamwork in the STEM fields utilize external resources to help female workers respond effectively to the pandemic.

## 1 Introduction

The COVID-19 pandemic has transformed the lives of people around the world, bringing about drastic changes and life-altering shifts to the workforce as a whole. Employers are facing significant challenges affected by the impact of the pandemic on multiple levels, such as the expansion of underemployment (1), the surge in employment pressure (2), and the increased need to balance work and family (3).

Akkermans et al. (4) claimed that the impact of the pandemic on careers is a product of the interaction between personal coping methods and the surrounding factors. Therefore, the effective coping strategy is to turn “crisis” into “opportunity.” Hite and McDonald (5) have suggested that coping strategies such as career resilience, developing multiple career skills, and enriching on-the-job training are all critical elements in developing a sustainable long-term career.

According to statistics from previous studies on the pandemic and careers, this research found that most of those studies examined the impact of the pandemic on the careers of the general public, and the related responses (2, 4, 6), while this research focused on a specific group of STEM (science, technology, engineering, and mathematics) female workers. There are two reasons why STEM females are chosen to be subjects of this research. (1) Previous studies have found that the proportion of STEM females is increasing. However, they still face more adversity than men (7), thus warranting more research efforts. (2) Previous studies have found that female employees are responsible for taking care of their families and are exposed to more family-work conflicts during the pandemic (3). It is worthwhile to investigate whether the work-from-home working type in the STEM field increases internal conflicts among female employees.

For reasons outlined above, this research was conducted with STEM females, aiming to understand the challenges they faced in their daily work during the pandemic, and the coping strategies they used.

## 2 Method

### 2.1 Participants

This research conducted purposive sampling (8) to invite research participants, and selected three female engineers who all worked in technology companies in Silicon Valley, California. The research participants all have master’s degrees; two participants (renamed as Illy and Candy) have more than 25 years of work experience and one participant (renamed as Yeh) has more than 3 years of work experience. The first two participants, Illy and Candy, aged between 50 and 55 and the third participant, Yeh was about 30–35 years old. I and C have their own marriages with two children, respectively. At the time of the study, they all work from home. Same as their husbands. All the children grow up and also work from home. The third participant Yeh is single.

### 2.2 Measures

Research tools used in this research included invitations, participation consent documents, interview outlines, and interview notes. The interview outline consists of four main parts: an introduction to the main content of the current work, the history of career development in the STEM field, the work difficulties encountered during the pandemic, and personal coping strategies.

### 2.3 Procedure

First, we determined the purpose of the research and then developed the interview outline. We then invited the participants to engage in an in-depth interview. Due to the pandemic, research interviews were conducted via Google meet or MS teams. Each interview lasted approximately 60–90 min. We then compiled and analyzed the data after the interviews and wrote this research report.

### 2.4 Qualitative analysis

Data compiling included two steps: (1) Writing a note after each interview to record the overall impression about participants. (2) Turning the audio file into a transcript, numbering the data, and anonymizing for privacy.

The data analysis was conducted through Content Analysis (9), which included four steps: (1) Repeatedly reading the transcript and supplementing with interview notes to outline the content related to the research topics. (2) Labeling the content related to the research topics as meaningful units. (3) Comparing the meaningful units, categorizing similar units, and naming them according to their connotations. (4) Comparing the associations between the topics, and categorizing them into broader themes.

## 3 Results

### 3.1 Quick responses to pandemic, new experience of working from home

The research participants indicated that in response to the pandemic, the work-from-home policy was implemented in a hastened manner. The advantage of working from home includes saving commute time, more freedom and flexibility during work hours, and the ability to take care of family life. Disadvantages of working from home include being forced to put hardware testing on hold, and your work may be affected by problems with the utilization of software and equipment. However, the research participants also pointed out that technology companies are high-tech industries and have demonstrated the ability to respond quickly. For example, technology companies quickly improved network systems, solved technical problems with network connections for working from home, and provided remote, timely, and personal assistance to employees. These quick responses not only solved the technical problems in the network connection of working from home, but also improved the efficiency of employees. Overall, after the pandemic, employees generally prefer to work from home, and reported being able to maintain their level of efficiency. The work of technology industries is also suitable for flexible working schedules.

“…When the pandemic started, the company’s network was not strong enough, and the network at home might not be stable as well. It often disconnected at the beginning, and I was very nervous at first. They (the company) specially arranged an IT person to help solve problems. There’s always an IT person available. When you need help in maintaining the machine or doing something else, IT would help you to do it immediately.” (I-36)

“The company has become very efficient in responding to these problems, and the whole company has become a work-from-home work unit, and everyone likes this instead.” (I-37)

The children of research participant C are also working in the technology field. They found that after the pandemic, young people preferred to work from home. In particular, young people want to travel to different places, and working from home allows them to work from different places.

“I’ve asked my kids who have a job, and they say that their peers who are looking for jobs now prefer those allowing them to work from home, because working from home allows them to travel…they are taking the opportunity to stay in different places…” (C-28)

### 3.2 New challenges of working from home

From the perspectives of the three participants, there are five challenges that STEM females experienced while working from home: time management, work efficiency, work and family balance, online teamwork maintenance, and adjustment to the environment.

#### 3.2.1 Time management

The most important feature of working from home is that it saves commute time and provides more flexibility in time arrangement. With a computer readily available, you can work whenever and wherever you want. However, this has also become a test of employees’ time management skills.

Research participants reported being allowed to schedule their own work hours at home. For example, participant I was able to incorporate life tasks, such as walking the dog and preparing meals, into her work intervals. In another example, participant C directly placed her desk near the kitchen to make the best use of spare minutes, such as preparing meals as the code is running. Participant Y, for example, found that working from home increased the amount of time being alone, which could be used for reading and thinking about future career planning.

“Now I put my office near the kitchen for work. I can wash the vegetables when running code or compare. For those (tasks) not completed, I can check it out around six or seven o’clock. It was originally a huge block, but now I can break the whole into parts. It seems to be a little more flexible.” (C-13-1)

However, the blurred boundaries between work and life caused by working from home can easily lead to overtime. For example, participant I pointed out that before the pandemic, she would actively avoid working overtime on weekends or in the middle of the night because of the need to commute or not having food in the middle of the night. However, working from home makes everything convenient, with a computer right beside you and food at your fingertips, it becomes easy to work when you need to, or to work overtime in times of urgency. Research participant C observed the way young people worked from home and found that they needed to be on call all the time, with no concrete distinction between being at work and leaving work.

“…In the past, if you wanted to do work on the weekend, you would feel troubled because you have to go into the office or something, and you would struggle a little bit and think, ‘Forget it, I’ll do it on Monday’. Nowadays, it’s most unlikely to be like this, and if you want to do your work, the computer is right next to you, so you can go anytime and start doing it.” (I-40)

“…Now my kids who do software are working from home, and he is, they are, always on. For example, when they have a problem, colleagues want to contact each other, he’s always on call, always online…” (C-29-1)

#### 3.2.2 Efficiency in work

Companies are most concerned about the efficiency of their employees working from home. Research participant I believed that productivity of working from home had not decreased but rather was higher than before, however, this increase in efficiency was caveated with the need for self-discipline. For example, if delaying work was necessary for avoiding disrupting a colleague’s vacation, Research participant I would need to establish a clear work schedule arrangement. Research participant Y had a different view. She thought that working from home was not only slower but also significantly less productive and had lower job satisfaction. Research participant Y believed that there were two reasons for the decrease in work efficiency: (1) Working from home did not allow for hardware testing, and regular work was easily interrupted by the pandemic. (2) Formal and informal brainstorming interactions between colleagues were not available due to the pandemic, and there was a lack of sources of creative inspiration, which are important factors for improving work efficiency.

“I think it (working at home during a pandemic) is more efficient than before.” (I-35)

“Working from home is not as efficient as usual. I’ll start with a few reasons, first, there’s less collaboration, and second is that… we need hardware to test, and in the case of a pandemic the testing becomes more complicated, which leads us to deliberately do more works related to software and less to hardware.” (Y-56)

#### 3.2.3 Work and family balance

The three research participants were unanimous in stating that working from home helps to balance their work and family. For example, research participant I found that working from home allowed her to schedule leisure activities in a convenient manner, such as walking the dog before returning to work or working while on vacation. Research participant Y also found that working from home was more comfortable and convenient, given that you can better handle things in your own home while working, such as renovating your house.

However, since work and family are shared in the same space, it can be a challenge to manage them simultaneously. For example, participant Y pointed out that it was convenient to eat and exercise when working in the office, while working at home required more effort to handle things such as cooking and cleaning. In another example, the chief executive of participant C left his job because of the stress of taking care of his children while working at home. It is evident that the task of balancing work and family life is a new challenge for people working from home.

“It’s like my former boss. He quit on his own because at that time his two kids were both on-line, and then the couple had to work. The kids were just in elementary school, so he had to spend time with them. They couldn’t sit still, which was very stressful for him, making him quit the job last June.” (C-35)

#### 3.2.4 Online teamwork maintenance

In the science and technology field, there is an emphasis on teamwork, and working from home transforms the original team communication into an online format. Regular formal online meetings would be arranged by the company, allowing employees to update their project progress. The staff also arranged informal online meetings to stimulate creativity through online discussions. For example, at the beginning of the pandemic, the efficiency of participant Y was affected by a lack of communication with colleagues. But as the team’s online communication increased, she found that online meetings could still have the same brainstorming benefits as before. The three research participants agreed that the outbreak had little impact on teamwork, with the only change of format to more frequent online meetings.

“…Basically, we have an update of your current progress every Monday, Wednesday, and Friday, so meetings are more frequent than before instead…” (I-35-2)

“Now the strategy is that we try to have more social time. We used to plan once every 2 weeks about our work, but now it has become once a week, more frequent, giving everyone the opportunity to communicate, formal and informal, and more channels to communicate.” (C-64)

#### 3.2.5 Adjustment in such an environment

The pandemic has hit the job market. The U.S. government has offered many vocational training projects to help the unemployed switch to new occupations, but there is still a need for active cooperation from the workforce. The three research participants agreed that American culture values freedom and autonomy, and that the government does not need to do much because people will find their own way out. For example, Research Participant I believes that the government had too much control and has done too much, and that it should give more freedom. In another example, research participant C found in her communication with young people that they are reconsidering the meaning of work. Some wanted to take a break from work, and some wanted to leave their jobs to start up their own businesses.

“In a free society like the United States, of course, individualism is still more important. It seems that the government doesn’t need to do anything, and people themselves will find opportunities to grow and keep their jobs going.” “Right, but I also know a person in the STEM field who said he didn’t find a suitable job, but he wanted to quit and emailed me at the end of last year, around Christmas. (C-30)

#### 3.2.6 Inner resources: the typical characteristics and beliefs of STEM women

While working from home, the STEM females we interviewed had a deeper view of their characteristics and career beliefs, which were inner resources to help them cope effectively with the pandemic. The results of the interviews show that STEM females have five major personal characteristics and beliefs: (1) Interest-oriented career choices, passion for STEM, and a sense of accomplishment from their jobs. (2) Emphasis on logical thinking, active learning, self-improvement, and a high sense of self-efficacy. (3) Enjoying innovation and adventure, embracing challenges, advocating freedom, and attaching importance to planning. (4) Being light on fame and fortune, reacting by nature, living in the present, and (5) Preferring relationships that are friendly, equal, and working together.

“I think the work now is quite interesting because every day there are some very challenging things or problems that no one has ever solved, and I would be very happy if I could solve them. I don’t think anyone is truly doing robots, intelligent robots, and I think our group is very likely to make it. Now the daily work is very challenging…” (Y-35-1)

### 3.3 External resources: corporate systems and teamwork in the technology field

Corporate systems and teamwork in the technology field are external resources that help STEM females respond effectively to the pandemic.

The three research participants unanimously pointed out that technology companies provide a free, open, stable, and fair environment, encourage autonomy and innovation, proactively help employees balance work and life, and provide substantial and consistent support. For example, research participant I shared that the company offers open space for creativity, excellent benefits, and there were little to no fear of layoffs. In another example, research participant Y shared that the company where she worked at was stable, paid well, and gave her enough freedom. There was no need to be afraid even if there were not any results in the short term.

“Because whenever you want to do some new work, new projects, the company will let you have a try, it won’t make you always do the same work. If you are willing to try new stuff, the company will let you do it. I think the company is stable, so you wouldn’t have that fear of being laid off…” (I-15)

Research participants I and Y mentioned that colleague relationships are generally friendly, balanced, understanding, and respectful. The team works together to achieve mutual success, which is crucial support for daily work. For example, participant I stated that team members could respect and accommodate each other. Research participant Y appreciated the teamwork and felt that her colleagues not only got along well with each other, but also demonstrated their talents, supported each other, and worked together, including actively participating in brainstorming to stimulate each other’s creativity and make contributions to each other’s achievements.

“I will propose some ideas, and then a good point is that colleagues are all nice, and then, there is no competition between coworkers in the workplace, people help each other to realize your idea.” (Y-35-2)

## 4 Discussion and implication

**Figure 1:**
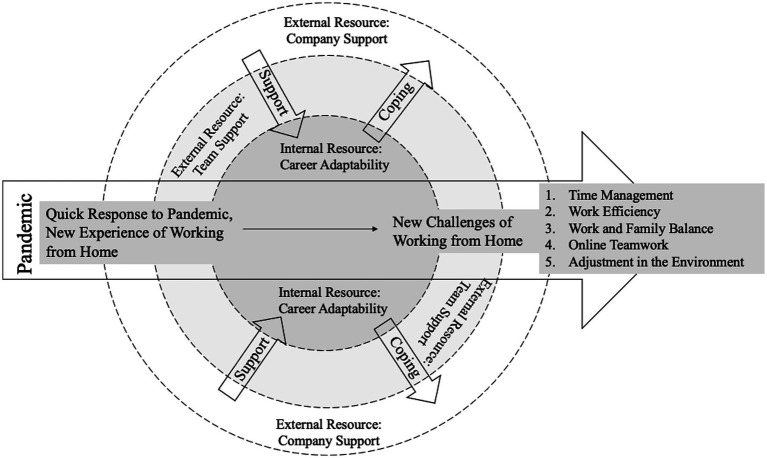
STEM female’s coping strategy under the COVID-19 pandemic.

Summarizing the above research results, this research integrated the career coping patterns of women in STEM fields during the pandemic into Figure 1. In response to the impact of the pandemic, technology companies responded quickly, and working from home has become the main working type. During the period of working from home, female workers experienced five challenges, including time management, work efficiency maintenance, work and family balance, and online teamwork development. In addition, in the face of the pandemic’s impact on the job market and the government’s multiple response measures, employees are trying to adjust themselves to help them settle into their work and family life in the environment. A proactive response to the pandemic cannot be achieved without both internal and external resources. The STEM females we interviewed found the company provided powerful backing, and team members also offered strong support. Meanwhile, female workers also found that the typical characteristics and beliefs of STEM females are inner resources that help them respond effectively to the pandemic. Overall, with the best use of inner resources and strong external support, STEM females have been able to minimize the impact of the pandemic on their daily work, and keep their work moving forward.

Although the pandemic has little impact on the day-to-day work routine for women working in STEM, this research did identify three important findings that are worthy of further discussion in follow-up research.

Firstly, this study found that all three women entered the field of science and technology based on their interests, and because of their high self-efficacy and excellent mathematical ability, they were identified to have a high sense of accomplishment stemming from their career. Schultheiss’s (7) maintains that interest, self-efficacy, and mathematical ability are important factors affecting employment in the field of science and technology. While our findings support these hypotheses, the study also further found that the influence of personal characteristics, beliefs, and values in effectively allowing women to face major environmental challenges such as the pandemic. These characteristics, beliefs, and values were identified as pivotal self-regulatory resources that assist in coping effectively with challenges. The results of this study also support the theoretical proposition of Savickas (10), that is, when faced with work difficulties, individuals will activate self-regulatory resources to actively cope with challenges. In other words, the unique personal characteristics, values, and beliefs of women in the field of science and technology are an important part of shaping women’s career adaptability.

Secondly, the three women unanimously emphasized the importance of external resources in coping with the impact of the pandemic, which is consistent with the findings of Lent et al. (11), where support systems are identified as a critical element affecting women’s performance in the STEM fields. In another study by Neo et al. (12), the findings suggested that family support is an important predictor of women’s career experience during the pandemic. To further expand on past research findings, this study found that the support received from tech companies and their respective teams also act as an important factor affecting women’s home office proficiency. Family support is always an important facilitator for females’ mental health.

Furthermore, this study found that STEM females who were currently in different career stages had various opinions regarding the efficiency of home office work. On the one hand, nearly all women in the tech industry believed that the boundaries between work and family were blurred due to the home office environment, and individuals needed to adjust and respond to maintain the balance between work and family. The results of this study are consistent with the findings of Neo et al. (12), that is, during the pandemic, the boundaries between work and family are blurred, and it has been quite challenging for female workers to balance work and family. On the other hand, consistent with the findings of Mockaitis et al. (6), the results of our study support that the pandemic affects workers at different stages of career development differently. Specifically, the two female employees in this study who were closer to the age of retirement believed that work efficiency only fluctuated at the beginning of the pandemic, but with the adjustment of the company and individual familiarization, the level of work efficiency was actually higher than being in the office. Female employees who are still at the beginning stages of their careers, however, believed that work efficiency has dropped significantly due to the pandemic, and they looked forward to more assistance to improve work efficiency. Regarding this finding, this study speculates that the soon-to-be-retired women, as senior women in STEM, may pay more attention to job stability and take job completion as the primary goal; while women who are just starting out aspire to pursue more challenges, desire to develop more work results, and are not as satisfied with the status quo. More challenges might be valued by young workers. The sense of success and accomplishment might be part of resources to promote their mental health and wellbeing (13).

Finally, the results of the interviews demonstrated that daily work of STEM females is mildly affected by the pandemic, this in part may be related to the U.S. work culture. As Guan et al. (14) argued in their study, people’s career lives in individualistic cultures are less affected by the pandemic than in collectivist cultures. Similarly, the three women selected for this study were Chinese-Americans who have worked in Silicon Valley for a long time. During the pandemic, their career coping has been deeply influenced by Western culture, emphasizing proactive response to external challenges and improving personal coping skills (14). The findings of this study also support the view of Akkermans et al. (4) where the impact of the pandemic on personal career is the product of the interaction between the individual and the situation. In addition, for the female STEM workers we interviewed, it seems that they can cope with the family–work conflict quite well under the pandemic. The main reason is that they do not have to drive to the office. Working from home saved a lot of time and money caused by transportation.

As far as the limitations of this study are concerned, due to the small number of samples in this study, the results of the study are still somewhat limited in the application of inference. Future research could be conducted in two directions: (1) working from home is more demanding in terms of balancing family and work life, which could be explored in more depth in future research; (2) working from home reduces face-to-face interpersonal communication, but increases online interaction. However, Riva et al. (15) found that there are still essential differences between online interaction and face-to-face communication, so future research can also further explore the impact of online interaction on routine work.

## Data availability statement

The raw data supporting the conclusions of this article will be made available by the authors, without undue reservation.

## Ethics statement

The studies involving humans were approved by Research Ethics Office, National Taiwan Normal University. The studies were conducted in accordance with the local legislation and institutional requirements. The participants provided their written informed consent to participate in this study.

## Author contributions

H-LT: Writing – original draft. LG: Writing – original draft. W-HW: Writing – original draft. JL: Writing – original draft.

## References

[1] Duffy RD Kim HJ Allan BA Prieto CG Perez G. Structural predictors of underemployment during COVID-19 pandemic: a psychology of working perspective. Couns Psychol. (2022) 50:477–505. doi: 10.1177/00110000221078819

[2] Autin KL Blustein DL Ali SR Garriott PO. Career development impacts of COVID-19: practice and policy recommendations. J Career Dev. (2020) 47:487–94. doi: 10.1177/0894845320944486

[3] Woodbridge LM Um B Duys DK. Women’s experiences navigating paid work and caregiving during the COVID-19 pandemic. Career Dev Q. (2021) 69:284–98. doi: 10.1002/cdq.12274, PMID: 35463741 PMC9015544

[4] Akkermans J Richardson J Kraimer ML. The Covid-19 crisis as a career shock: implications for careers and vocational behavior. J Vocat Behav. (2020) 119:103434. doi: 10.1016/j.jvb.2020.103434, PMID: 32390655 PMC7205633

[5] Hite LM McDonald KS. Careers after COVID-19: challenges and changes. Hum Resour Dev Int. (2020) 23:427–37. doi: 10.1080/13678868.2020.1779576

[6] Mockaitis AI Butler CL Ojo A. COVID-19 pandemic disruptions to working lives: a multilevel examination of impacts across career stages. J Vocat Behav. (2022) 138:103768. doi: 10.1016/j.jvb.2022.103768, PMID: 35999896 PMC9388277

[7] Schultheiss DE. The role of gender in career development In: Brown SD Lent RW, editors. Career development and counseling: Putting theory and research to work. 3rd ed. Hoboken, New Jersey: John Wiley & Sons (2020). 273–308.

[8] Maxwell JA. Qualitative research design: An interactive approach. 3rd ed Thousand Oaks, California: SAGE (2013).

[9] Bengtsson M. How to plan and perform a qualitative study using content analysis. NursingPlus Open. (2016) 2:8–14. doi: 10.1016/j.npls.2016.01.001

[10] Savickas ML. The theory and practice of career construction In: Brown SD Lent RW, (editors) Career development and counseling: Putting theory and research to work. 3rd ed. Hoboken, New Jersey: John Wiley & Sons (2020). 165–199.

[11] Lent RW Sheu H-B Miller MJ Cusick ME Penn LT Truong NN. Predictors of science, technology, engineering, and mathematics choice options: a meta-analytic path analysis of the social–cognitive choice model by gender and race/ethnicity. J Couns Psychol. (2018) 65:17–35. doi: 10.1037/cou0000243 29355343

[12] Neo LS Tan JYC Chew TWY. The influence of COVID-19 on women’s perceptions of work-family conflict in Singapore. Soc Sci. (2022) 11:73. doi: 10.3390/socsci11020073

[13] Whealin JM Saleem JJ Vetter B Roth J Herout J. Development and cross-sectional evaluation of a text message protocol to support mental health well-being. Psychol Serv. (2023) 20:657–67. doi: 10.1037/ser0000601, PMID: 34968123

[14] Guan Y Deng H Zhou X. Understanding the impact of the COVID-19 pandemic on career development: insights from cultural psychology. J Vocat Behav. (2020) 119:103438. doi: 10.1016/j.jvb.2020.103438, PMID: 32382162 PMC7204647

[15] Riva G Wiederhold BK Mantovani F. Surviving COVID-19: the neuroscience of smart working and distance learning. Cyberpsychol Behav Soc Netw. (2021) 24:79–85. doi: 10.1089/cyber.2021.0009, PMID: 33577414

